# Continuous Exposure to Ethylene Differentially Affects Senescence in Receptacle and Achene Tissues in Strawberry Fruit

**DOI:** 10.3389/fpls.2020.00174

**Published:** 2020-03-12

**Authors:** Roberta Tosetti, Fardusa Elmi, Inmaculada Pradas, Katherine Cools, Leon A. Terry

**Affiliations:** Plant Science Laboratory, Cranfield University, Bedfordshire, United Kingdom

**Keywords:** abscisic acid homeostasis, invertase, non-climacteric fruit, senescence precursor, ethylene

## Abstract

Strawberry shelf life is limited, and little is known about the postharvest regulation of senescence in different fruit tissues. Strawberry is classified as a non-climacteric fruit, yet it is known that ethylene affects strawberry ripening. Here the effects of continuous exogenous ethylene (50 µl l^−1^) were investigated in cold stored strawberry (5°C). The physiological and biochemical responses of ripe strawberry were evaluated across 6 days, together with hormonal profiles of the whole fruit and individual tissues (achenes and receptacle). Continuous exposure to ethylene induced as a first response an accumulation of abscisic acid (ABA) in the receptacle tissue, followed by an increase in CO_2_ production. Ethylene also elicited sucrose hydrolysis and malic acid catabolism, with the major effect seen after 4 days of ethylene exposure. Additionally, accumulation of phenolics (epicatechin and chlorogenic acid) were also observed in ethylene treated strawberry. Achenes did not exhibit a response to ethylene, yet catabolism of both ABA and auxins increased by two thirds during air storage. In contrast, ethylene induced ABA accumulation in the receptacle tissue without ABA catabolism being affected. This hormonal disequilibrium in response to ethylene between the two tissues was maintained during storage, and therefore might be the precursor for the following biochemical variations reported during storage.

## Introduction

The high perishability of strawberry represents a bottleneck for expanding the fresh market supply chain (Terry et al., 2011). Strawberry is classified as a non-climacteric fruit and its ripening is mainly regulated by the abscisic acid (ABA)/auxins ratio (Moya-León et al., 2019). In particular, auxins have been associated with ripening inhibition, while ABA accumulation has been linked with promotion of strawberry fruit ripening (Jia et al., 2011; Symons et al., 2012; Leng et al., 2014; Chen et al., 2015; Chen et al., 2016; Giné-Bordonaba and Terry, 2016; Jia et al., 2016; Li et al., 2016b; Medina-Puche et al., 2016). Despite this, an increasing number of studies have highlighted that ethylene does affect the late stages of ripening (Gu et al., 2019). Confirmations of ethylene involvement in strawberry ripening are also supported by research using the ethylene binding inhibitor, 1-methylcyclopropene (1-MCP) (Ku et al., 1999; Jiang et al., 2001; Bower et al., 2003; Villarreal et al., 2010; Villareal et al., 2016).

Ethylene plays a key role in the development of strawberry color, in the accumulation of taste-related compounds (flavonoids, phenolics, organic acids, and sugars), and in the softening process (Villarreal et al., 2010; Merchante et al., 2013; Li et al., 2016a; Villareal et al., 2016; Moya-León et al., 2019). Moreover, it has been reported that ethylene biosynthesis and signaling genes are differentially expressed during ripening of strawberry, and that some Ethylene Response Factors (ERFs) showed an expression pattern similar to those found in a climacteric fruit (Trainotti et al., 2005; Sun et al., 2013; Sánchez-Sevilla et al., 2017). Nowadays, it is well understood that ripening involves cross-talk among different hormones rather than single hormone action (Chang et al., 2013; Chen et al., 2015). However, the complexity of ripening regulation in strawberry is also attributable to the peculiar anatomy of the fruit, which is composed of achenes (true fruit) and a swollen fleshy receptacle. The two tissues are different in terms of origin, physiological and biochemical roles, and metabolic networks (Fait et al., 2008; Csukasi et al., 2011; Symons et al., 2012; Gu et al., 2019). It has been demonstrated that endogenous ethylene production in ripening strawberry is mainly attributable to the achenes (Iannetta et al., 2006). Nevertheless, a recent study describing the transcriptional and hormonal profiles of the two tissues highlighted that ethylene related genes were upregulated in the receptacle only (Sánchez-Sevilla et al., 2017). Strawberry ripening seems therefore to depend on how the achenes and receptacle differentially respond and synergistically interplay with one another (Merchante et al., 2013).

Despite increasing knowledge about strawberry ripening, it is surprising that little work has evaluated the postharvest hormonal regulation of strawberry physiology. Previous evidence has suggested that ethylene and the control thereof can have both positive and negative effects during postharvest storage of strawberry (Terry et al., 2007a), but the interplay between exogenous ethylene and ABA during postharvest is still unknown. One of the consequences of this paucity of information is also reflected in the limitation of strawberry shelf life management strategies.

To better characterise the role of ethylene, the postharvest effects of continuous exogenous ethylene (50 µl l^−1^) exposure were investigated. The physiological and biochemical responses of ripe strawberry were evaluated across 6 days of cold storage, together with the hormonal profiles of whole fruit and individual tissues (achenes and receptacle).

## Materials and Methods

### Plant Material and Ethylene Treatment

Ripe strawberry fruit (cv. Sonata, n = 252) were purchased from a local grower (H & H Duncalfe in Cambs., UK). Plants were grown under standard commercial conditions (Spanish tunnels), and fruit harvested on 6th August before being transferred to Cranfield University within 2 h. On arrival, disease-free fruits of similar size and weight were selected, placed in plastic punnets and subjected to two different storage environments: continuous air (control), continuous ethylene supplementation (50 µl l^−1^). Each punnet contained n = 12 fruit and for each treatment there were n = 5 punnets (n = 60 fruit per treatment). The punnets were placed inside labeled 13 L sealed plastic boxes stored at 5°C for 6 days to simulate shelf life conditions in a domestic refrigerator, and constantly flushed to avoid the creation of modified atmosphere. Continuous exposure to ethylene was achieved by flushing ethylene in air (50 µl l^−1^, 250 ml min^−1^) using a blower manifold (Amoah et al., 2017) (custom built and supplied by Air Equipment, Beds., UK). After 2 h from the beginning of treatment the concentration of ethylene (50 µl l^−1^) was quantified as previously described (Terry et al., 2007a). Control strawberries were constantly flushed with cleaned air (250 ml min^−1^) from a cylinder of pure air to ensure complete air exchange within the boxes and to prevent the creation of a modified atmosphere.

### Fruit Sampling

At each sampling time (0 d; days of storage, 1 d, 2 d, 4 d, 6 d), the 13 l boxes were opened for few seconds and 12 fruit from each treatment (n = 12) were collected. They were separated into three blocks (n = 4 fruit) and assessed for CO_2_ and ethylene production, weight loss and color changes. Each of these parameters was assessed on three replicates per treatments. After the physiological measurements, the fruit were longitudinally halved. Following division, fruit were immediately snap frozen and freeze dried for further analysis. One half was used as whole fruit and the other was divided into achenes and receptacle tissues (Terry et al., 2007b). The receptacle tissue and achenes were manually separated from freeze dried material, using a pair of tweezers. All the achenes from four halves of strawberry fruit per treatment (* 3 blocks) were collected at each time point. Fruit assessed on day zero, before continuous ethylene exposure, were used for baseline measurements.

### Physiological Assessments

#### CO_2_ Production Measurement and Weight Loss

Real time CO_2_ production rate of strawberries were measured *in situ* (5°C) connecting the 13 l boxes to a Sable Respirometer System (Model 1.3.8 Pro, Sable Systems International, NV, USA) according to Alamar et al. (2017). The blocks (four fruits) for each treatment were weighed before each measurement. The weight was used to calculate the CO_2_ production per kg of fruit, and the percentage of weight loss.

#### Ethylene Production Rate

Real time ethylene production was continuously monitored (for 3 h) *ex situ* (20°C) with a laser-based photoacoustic ethylene detector ETD-300 (Sensor Sense B.V., Nijmegen, The Netherlands) as previously described (Salman et al., 2009).

#### Color Measurements

Objective color (lightness; L*), chroma index (color saturation; C*), hue angle (H°) was determined for each fruit (n = 12) per treatment as previously reported (Terry et al., 2007a). Measurements were obtained using a Minolta CR-400 colorimeter and DP-400 data processor (Minolta Co. Ltd., Japan).

### Biochemical Analysis

#### Sugars and Organic Acids Extraction and Quantification

Sugars and organic acids were extracted from whole fruit samples as previously reported (Terry et al., 2007a) and quantified using an Agilent 1200 series HPLC binary pump system (Agilent, Berks., UK) coupled with either an Agilent refractive index detector (RID) G1312A for sugars or a G1364C/G1315D photodiode array for organic acids. The concentration of glucose, fructose and sucrose, and citric, ascorbic and malic acids were calculated by comparison against an external calibration curve prepared with authentic standards (Sigma-Aldrich, Dorset, UK) and the results expressed as g kg^−1^ of dry weight.

#### Phenolic Compounds Extraction and Quantification

Phenolic compounds were extracted from whole fruit samples as previously described (Terry et al., 2007b). Concentrations of total anthocyanins, pelargonidin-3-glucoside, pelargonidin-3-glucoside derivative, cyanidin-3-glucoside, quercetin-3-glucoside, catechin, epicatechin, ellagic and chlorogenic acids (Sigma-Aldrich, Dorset, UK or Extrasynthese, Lyon, France) were calculated against authentic standards and expressed as mg kg^−1^ of dry weight.

### Extraction and Quantification of Strawberry Phytohormones

Phytohormones were extracted from both whole fruit and individual tissues (achenes and receptacle) and analyzed as previously described (Ordaz-Ortiz et al., 2015). An Agilent 6540 Ultra High Definition Accurate Mass Q-TOF LC-MS System was used to quantify abscisic acid (ABA), 7'OH-abscisic acid (7'OH-ABA), indole-3-acetic acid (IAA) and indole-3-acetylaspartic acid (IAAsp). Calibration curves for the four compounds were generated by plotting the known concentration of the calibration level (ABA and 7'-OH ABA were purchased from National Research Council of Canada-Plant Biotechnology Institute, Ottawa, Canada. IAA and IAAsp were purchased from OlchemIm Ltd, Olomouc, Czech Republic) against the relative response calculated for each calibration level. Quantification analysis was carried out using an Agilent MassHunter Quantitative Analysis Software B.05.00. Endogenous phytohormones concentration was calculated as µg kg^−1^ of dry weight.

### Statistical Analysis

Statistical analysis of results was performed using STATISTICA software for Windows (Dell Inc., 2015. Dell Statistica, version 12). Data were checked for residuals distribution, and tested with ANOVA, followed by a comparison of the means according to a least significant difference (LSD) test at *p <* 0.05.

## Results

### Physiological Assessments

**Figure 1:**
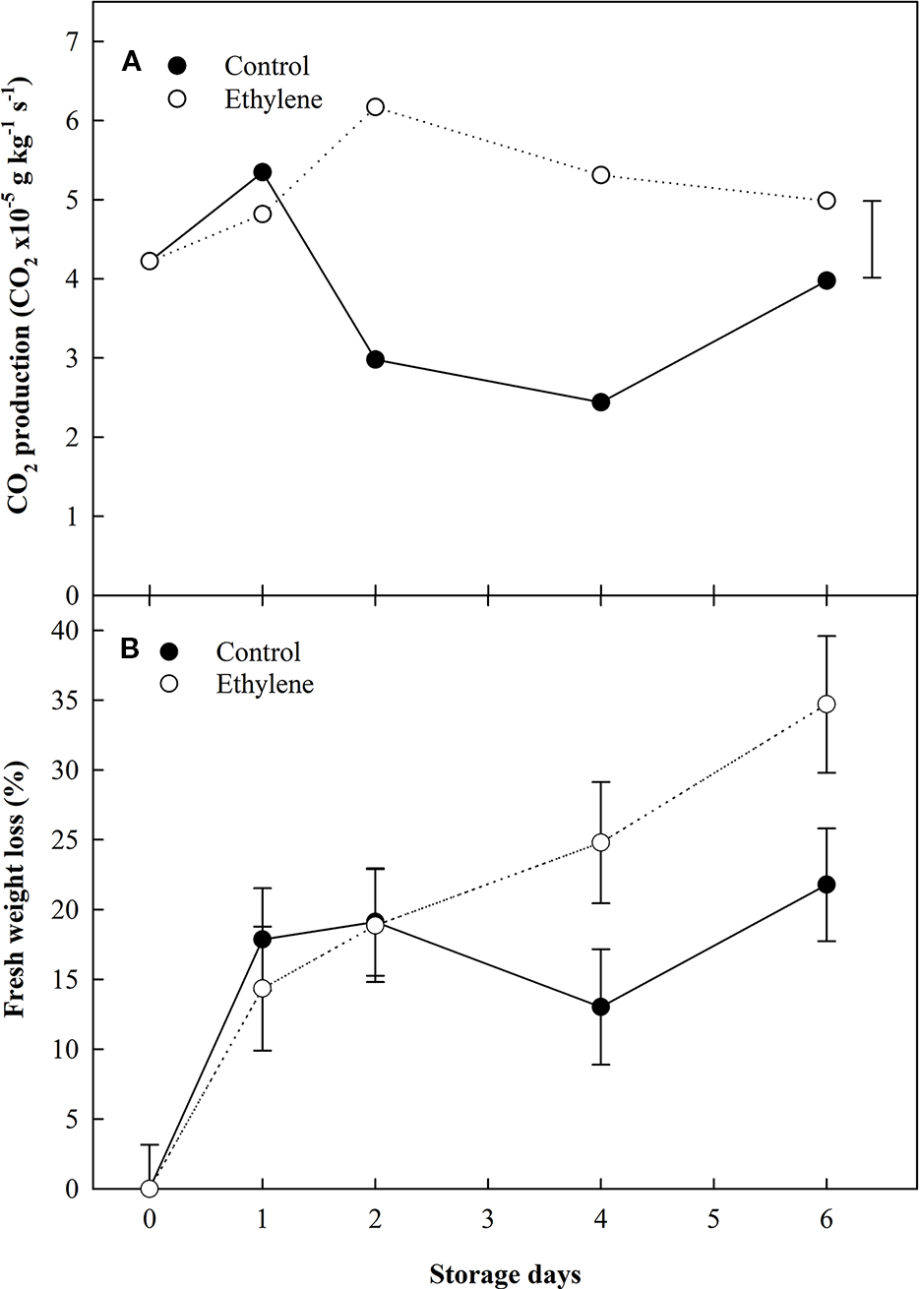
The effect of ethylene (continuous exposure) on CO_2_ production (CO_2_ × 10^×5^ g kg^−1^ s^−1^) and fresh weight variation (kg) of strawberries, cv. Sonata, stored at 5°C for 6 d. **(A)** CO_2_ production. Vertical bar represents the LSD (p <0.05). **(B)** Fresh weight variation. Vertical bars represent the Standard Error.

The physiological parameters of respiration activity in terms of CO_2_ production, weight loss and color changes of strawberry fruit were assessed. Significant variations relating to continuous ethylene exposure were identified in the production of CO_2_ and weight loss (**Figure 1**), while color parameters were more affected by the storage time.

Ethylene treated fruit exhibited a more than 2-fold increase in CO_2_ production compared to the control fruit during the storage (**Figure 1A**). However, by the end storage (6 d) the CO_2_ production of control fruit was similar to ethylene treated fruit. The ethylene treated fruit showed a total weight loss of circa 35% at the end of storage, while the difference in weight in control fruit was around 20% (**Figure 1B**).

The color evolution assessment showed that the chroma index (C*) and the hue angle (H°) decreased during the storage and exhibited a more marked decline in ethylene treated fruit. However, significant differences were identified only in relation with the storage day (*p <* 0.0000 and *p <* 0.0052, respectively) (**Table 1**), while the lightness (L*) did not change (*data not shown*).

**Table 1:** The effect of ethylene (continuous exposure) on chroma index (C*) and the hue angle (H°) of strawberries, cv. Sonata, stored at 5°C for 6. d. LSD (p < 0.05) for the overall means of the significant variable “storage day”.

Storage day	C*			H°		
	Control	Ethylene	LSD	Control	Ethylene	LSD
**0**	48.22			36.13		
**1**	51.04	51.63		37.16	37.88	
**2**	47.89	49.57		34.26	36.55	
**4**	47.59	45.61		35.75	34.24	
**6**	48.34	44.30		35.24	34.84	
			2.24			1.58

### Biochemical Changes Induced by Continuous Ethylene Exposure: Sugars/Organic Acids, and Phenolics Compounds

**Figure 2:**
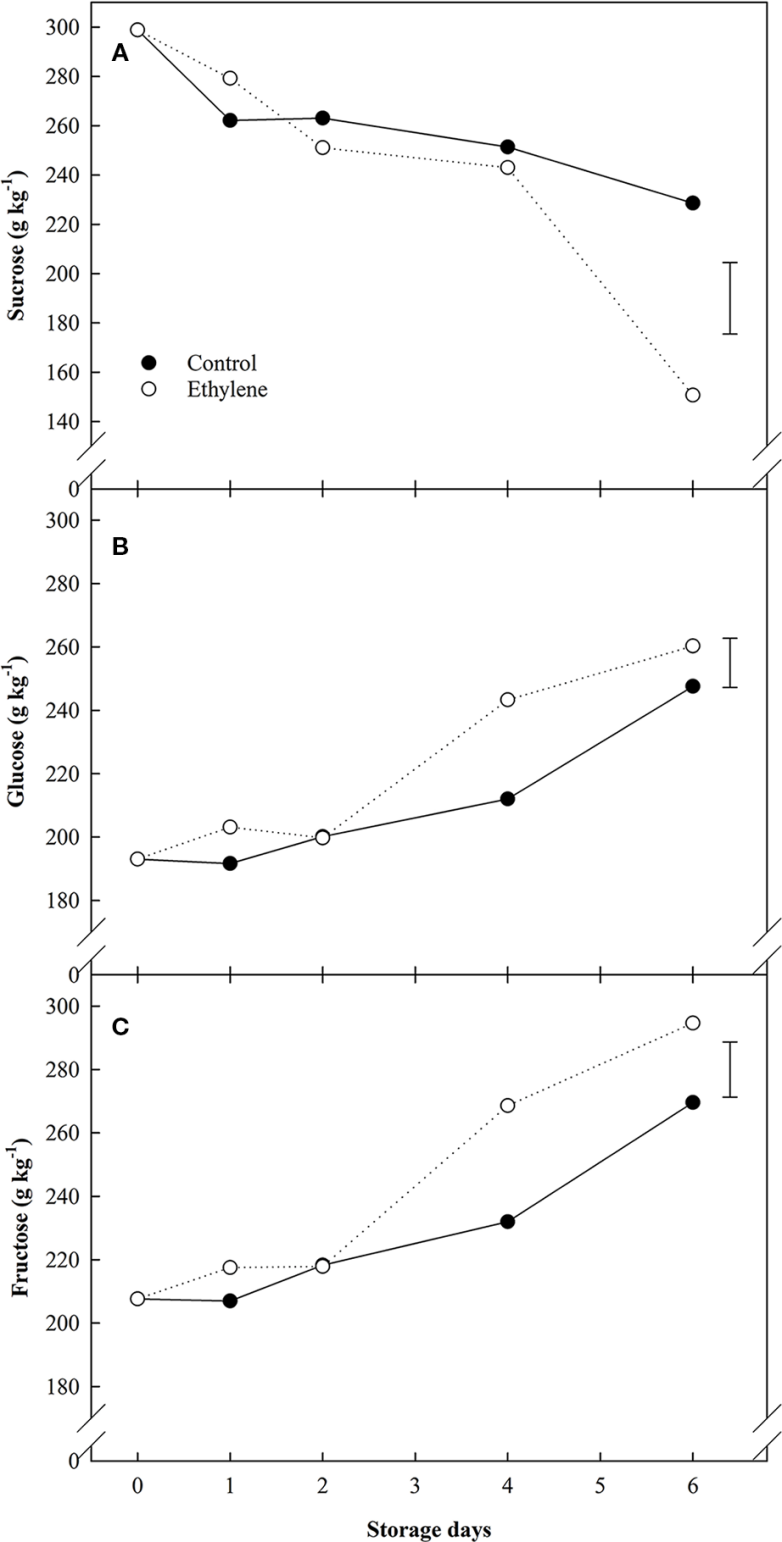
The effect of ethylene (continuous exposure) on sugars content (g kg^−1^) of strawberries, cv. Sonata, stored at 5°C for 6 d. **(A)** Sucrose content. **(B)** Glucose content. **(C)** Fructose content. Vertical bar represents LSD (p <0.05) for the overall means of the significant variable “treatment”.

Sugars data showed significant differences related to both treatment and storage day (**Figure 2**). Ethylene induced sucrose hydrolysis and accumulation of reducing sugars. In particular, ethylene treated strawberries showed increased sucrose hydrolysis from day 4 of storage, and by 6d, ethylene treated fruit had halved their initial sucrose values. In contrast, sucrose concentrations in control fruit decreased by *ca*. 25% compared to the initial amounts (**Figure 2A**). Concomitantly, ethylene-treated also induced the accumulation of reducing sugars in treated fruit (glucose 1.05-fold increase, fructose 1.1-fold increase) (**Figures 2B, C**, respectively).

**Figure 3:**
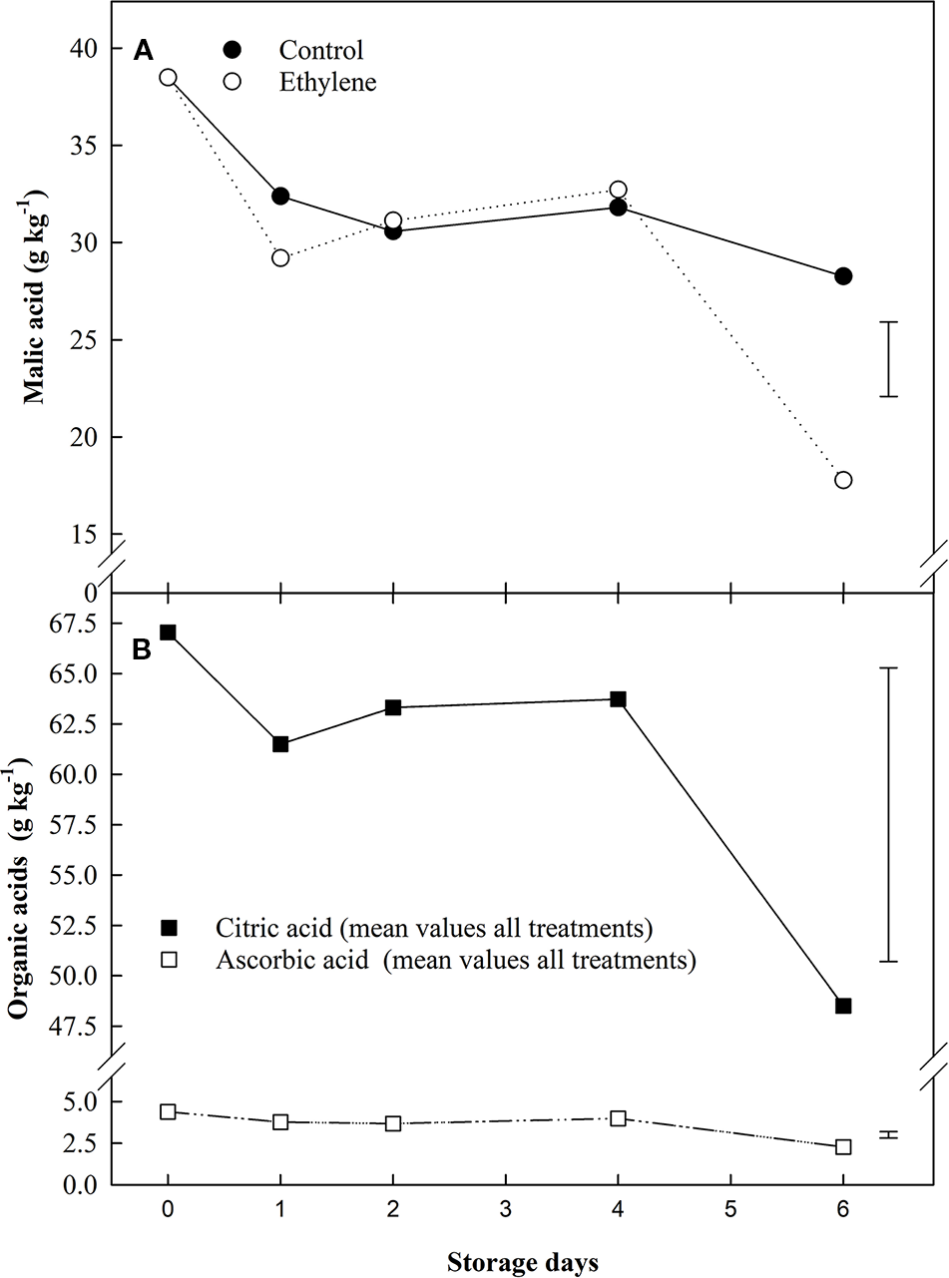
The effect of ethylene (continuous exposure) on organic acids content (g kg^−1^) of strawberries, cv. Sonata, stored at 5°C for 6 d. **(A)** Malic acid content (g kg^−1^) of strawberries, cv. Sonata, stored at 5°C for 6 d. Vertical bar represents LSD (p < 0.05) for the overall means of the significant variable “treatment”. **(B)** Ascorbic and citric acids. The represented values show the mean of all treatments (control and ethylene treated fruit) content. ■ citric acid. □ ascorbic acid. Vertical bar represents LSD (p < 0.05) for the overall means of the significant variable “Storage day”.

Of the organic acids analyzed, ethylene only had a significant impact on malic acid, and this effect was similar to the response for sucrose in treated fruit (**Figure 3A**). Citric and ascorbic acid content were affected by the storage time only (**Figure 3B**). At the beginning of storage, the most abundant organic acid was citric acid (67 g kg^−1^), followed by the malic (38 g kg^−1^), oxalic (6.7 g kg^−1^) and ascorbic acid (4.3 g kg^−1^), respectively; despite these variations in initial organic acids levels, an overall decline was observed at the end of the storage.

Total anthocyanins and three specific anthocyanins, cyanidin-3-glucoside, pelargonidin-3-glucoside, and pelargonidin-3-glucoside derivative, were analyzed. Significant differences were identified for total anthocyanins and pelargonidin-3-glucoside content, and were due to the storage day only (*p <* 0.0203 and *p <* 0.0047, respectively). Both compounds *ca*. increased 1.1-fold during storage without marked differences between ethylene and air (**Supplemental Figure 1**).

**Figure 4:**
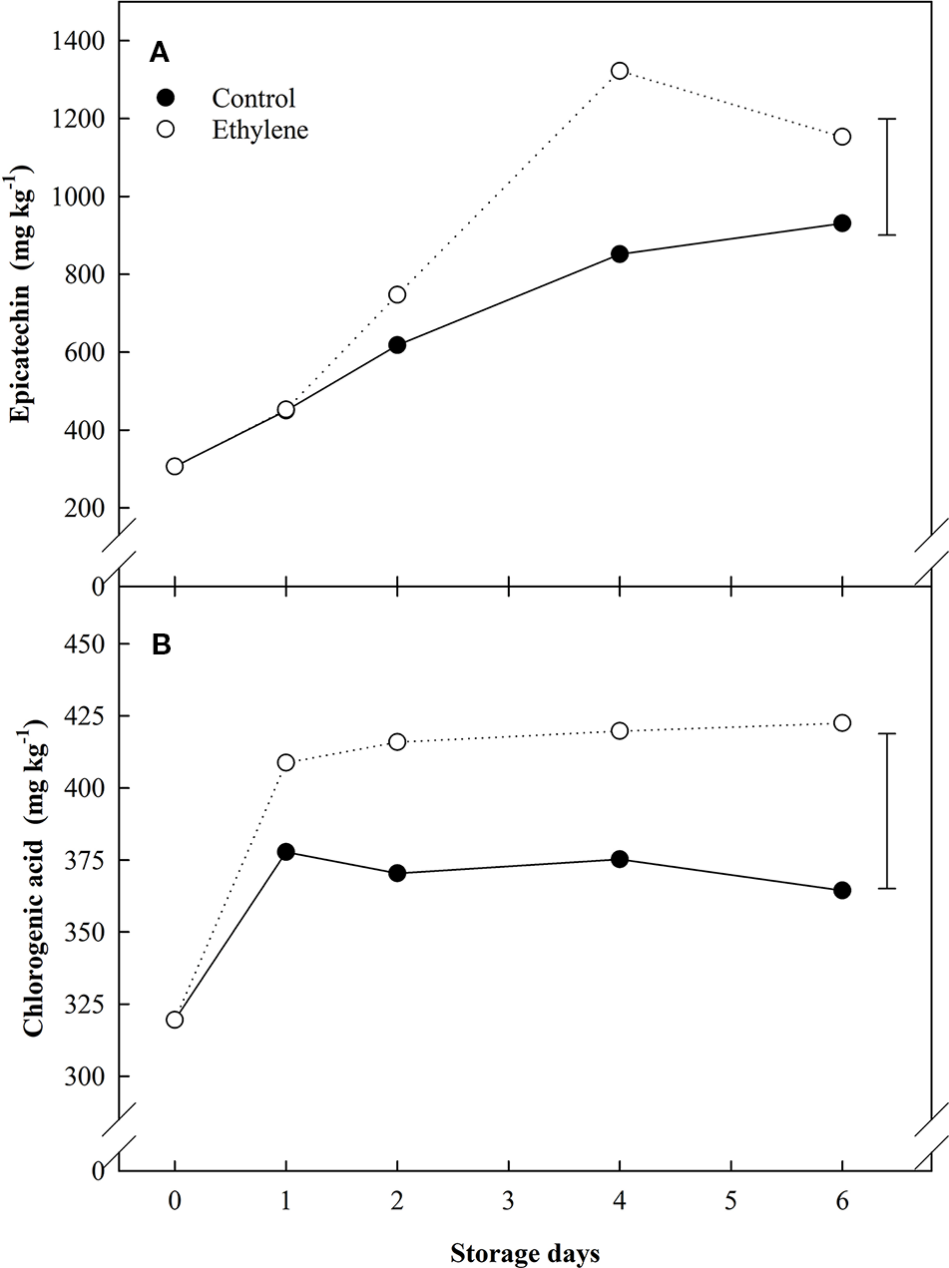
The effect of ethylene (continuous exposure) on phenolics content (mg kg^−1^) of strawberries, cv. Sonata, stored at 5°C for 6 d. **(A)** Epicatechin. **(B)** Cholorogenic acid. Vertical bar represents LSD (p < 0.05) for the overall means of the significant variable “treatment”.

Besides anthocyanins, other phenolic compounds were also quantified (quercetin-3-glucoside, catechin, epicatechin, chlorogenic and ellagic acids). Among them, quercetin-3-glucose did not show any significant difference; catechin and ellagic acid showed quite steady levels during all the storage (400–450 mg kg^−1^ and 35.8–36.1 mg kg^−1^, respectively. *Data not shown*). In contrast, epicatechin and chlorogenic acid levels were significantly affected by ethylene supplementation (**Figure 4**). Ethylene induced a gradual accumulation of epicatechin in the first four days of ethylene exposure (**Figure 4A**), while a sudden increase was observed for chlorogenic acid from day 1 until the end of storage (50% more than untreated fruit) (**Figure 4B**).

### Phytohormones

The effect of continuous ethylene exposure on phytohormone profile was studied. The *ex-situ* (20°C) monitoring of endogenous ethylene production showed a significant difference only according to the storage time, and overall the endogenous ethylene production increased during storage by 2.6-fold (**Supplemental Figure 2**).

**Figure 5:**
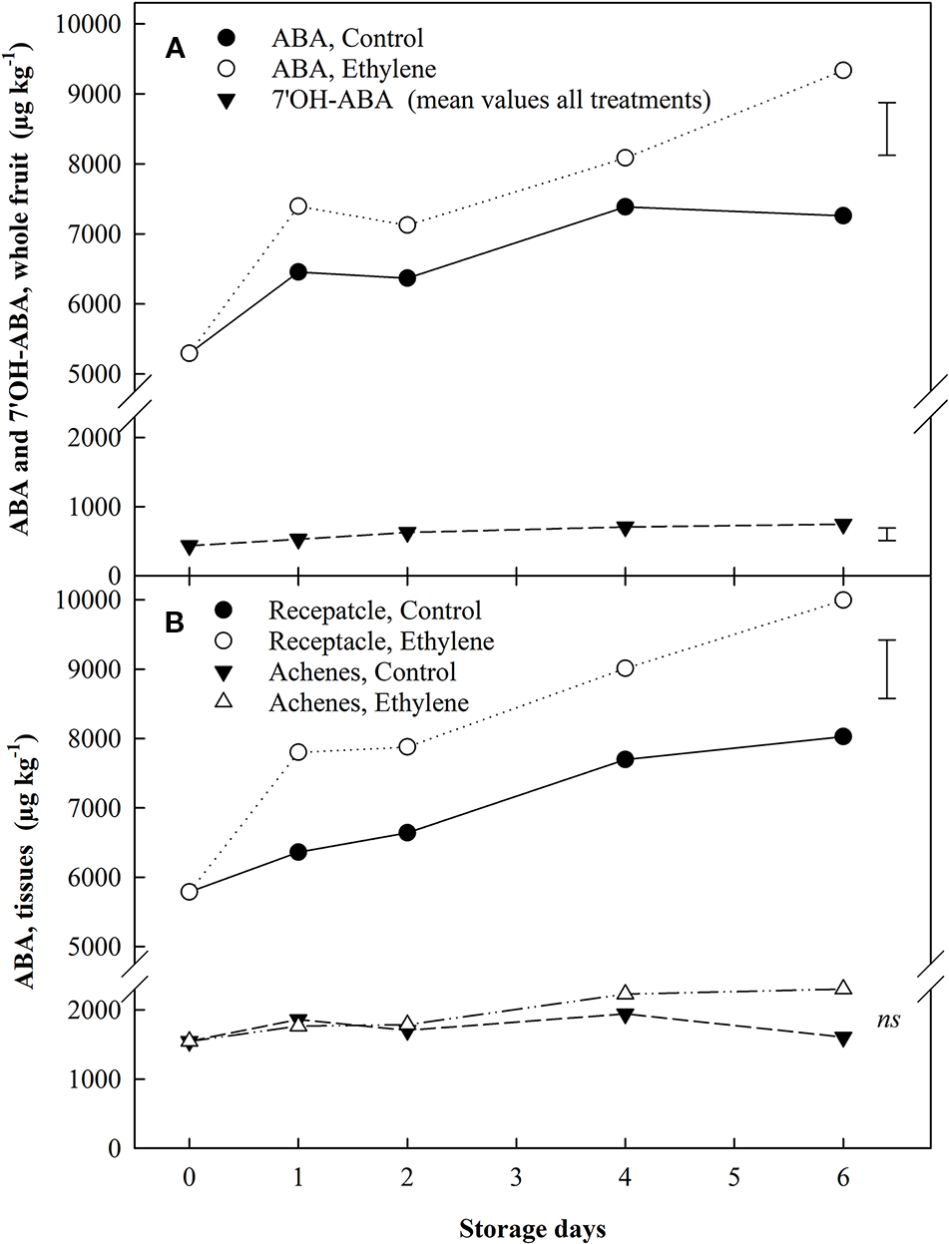
The effect of ethylene (continuous exposure) on ABA and the metabolite 7'OH-ABA content (µg kg^−1^) of strawberries, cv. Sonata, stored at 5°C for 6 d. **(A)**, ABA and the metabolite 7'OH-ABA content in the whole fruit. For the metabolite 7'OH-ABA the represented values show the mean of all treatments (control and ethylene treated fruit) content. **(B)**, ABA in individual tissues. Vertical bar represents LSD (p < 0.05) for the overall means of the significant variable “treatment” for ABA in both graphs and of the significant variable “Storage day” for 7'OH-ABA (whole fruit); ns, not significant.

**Figure 6:**
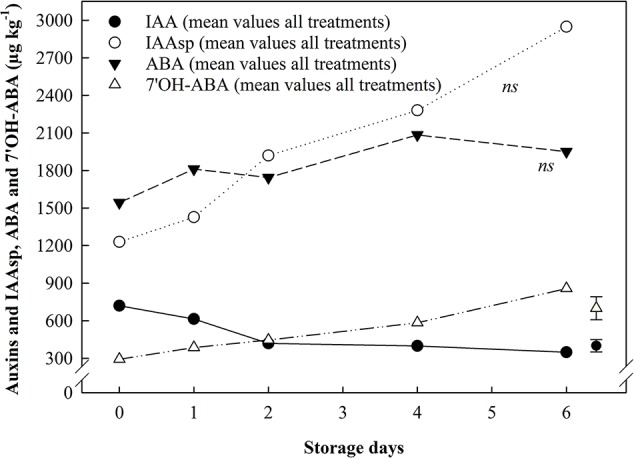
The effect of ethylene (continuous exposure) on auxins and ABA, and their catabolites, content (µg kg^−1^) in achenes of strawberries, cv. Sonata, stored at 5°C for 6 d. The represented values show the mean of all treatments (control and ethylene treated fruit) content. Vertical bar represents LSD (p <0.05) for the overall means of the significant variable “Storage day” for IAA and 7'OH-ABA; ns, not significant.

The quantification of two phytohormones and two metabolites thereof [abscisic acid (ABA), 7'OH-abscisic acid (7'-OHABA), indole-3-acetic acid (IAA), indole-3-acetylaspartic acid (IAAsp)] was carried out, using LC-MSMS, in the whole strawberry fruit as well as in individual tissues. Continuous ethylene exposure increased ABA content in the whole fruit and in the receptacle throughout storage (**Figure 5A**). ABA content was 4-times higher in receptacle tissue (only) than achenes at the beginning of storage, and the ethylene-induced ABA increase in the receptacle showed a steady difference of *ca*. 1.2-fold from day 1 in comparison with the untreated fruit content (**Figure 5B**). Achenes did not exhibit a response to ethylene, yet catabolism of ABA and auxins increased by two thirds during air storage (**Figure 6**).

7'OH-ABA content was more affected by storage duration than by ethylene treatment. An overall increase in 7'OH-ABA was found in all the tissues analyzed. 7 OH-ABA levels in whole fruit showed a mean increase of 1.71-fold (**Figure 5A**), while in the receptacle tissues no significant differences were identified (*data not shown*). In the achenes, 7'OH-ABA exhibited *ca*. 3-fold increase compared to the beginning of storage (**Figure 6**).

Auxins (IAA and IAAsp) content were detected only in the achenes, since the concentration of these phytohormones in the whole fruit and in the receptacle was below the quantification limit. IAA levels showed significant variations relating to storage days only. An overall 2-fold decrease in IAA content was reported across storage, although the major changes happened during the first two days. IAAsp exhibited an increasing trend (*ca*. 2.4-fold) in both control and ethylene treated fruit but a significant variation was not identified (**Figure 6**).

## Discussion

Previous studies investigating the effect of high level of ethylene (100 µl l^−1^) did not report a clear mechanism of response in cold stored strawberry (Siriphanich, 1980; Terry et al., 2007a). As consequence, it has been suggested that high concentration of ethylene would not impact on strawberry shelf life, while very low concentration (<1 µl l^−1^) have been reported to promote decay and softening (Wills and Kim, 1995). To better understand whether and how high level of ethylene can affect the postharvest quality of strawberry, the current work describes the responses induced by continuous ethylene exposure (50 µl l^−1^) in cold stored ripe strawberry.

Strawberry is classified as a non-climacteric fruit even though an increasing number of studies highlight that the receptacle exhibits some ripening features similar to a climacteric fruit. Although strawberry does not show the typical ethylene climacteric rise in respiration, it has been reported that ethylene biosynthesis and signaling pathways are differentially affected during strawberry ripening, and these changes are mainly localized in the receptacle (Trainotti et al., 2005; Iannetta et al., 2006; Merchante et al., 2013; Sun et al., 2013; Sánchez-Sevilla et al., 2017). Besides this transcriptional activation, the role of ethylene in modulating sugars/organic acid ratio, promoting anthocyanins accumulation and softening during strawberry ripening has been reported (Villarreal et al., 2010; Merchante et al., 2013; Villareal et al., 2016; Gu et al., 2019; Moya-León et al., 2019). Ethylene has been linked with increased CO_2_ production and water loss in ripening strawberry fruit (Tian et al., 2000); this being in agreement with results herein. Opazo and colleagues (2013) reported differential effects of ethylene and 1-MCP on the expression of the softening-related gene xyloglucan endotransglycosylase/hydrolase 1 (*XTH1*), due to the presence of ethylene responsive elements in the promoter region. The finding was confirmed by Villareal and colleagues (2016) who described the response to ethylene of several strawberry cell-wall metabolic genes. It has also been extensively reported that ethylene treatment elicited anthocyanins accumulation in ripening strawberry (Ku et al., 1999; Bower et al., 2003; Villarreal et al., 2010; Merchante et al., 2013; Sun et al., 2013; Lopes et al., 2015). In addition, Sun and colleagues (2013) demonstrated that the expression of *FaSAMS1*(S-adenosyl-L-methionine synthase 1) and *FaCTR1* (constitutive triple response 1) genes played a significant role in the softening and red-color development of strawberry fruit. The results herein did not show any effect of ethylene exposure on anthocyanins content; this difference may be explained since the fruit used in the current study were already at red ripe stage. In addition, the fruit were kept in cold storage, rather than 20°C, as indicated in previous works.

Lopes and colleagues (2015) reported that ethephon treatment increased sugars content in strawberry fruit both when applied in the field, and during postharvest. The results herein confirmed the propensity of ethylene to elicit an accumulation of reducing sugars, together with a concomitant decline in sucrose content during postharvest, as previously described (Li et al., 2019). In blueberry, ethylene can positively influence sucrose metabolism and invertase enzymes activity (Wang et al., 2020), and Jia and colleagues (2016) reported the key role of acid invertase during strawberry ripening. This may explain the activation of sucrose hydrolysis described here. Nevertheless, the taste and quality of strawberry fruit are linked to the sugars/organic acids ratio rather than sugars content alone (Giné-Bordonaba and Terry, 2009). Although both citric and malic acids are important compounds influencing strawberry acidity, malic acid is the main acid responsible for changes in pH regulation (Fait et al., 2008). The data of this work showed that malic acid catabolism was positively affected by continuous ethylene exposure, and developed concomitantly with the sucrose decline. These similar variations of malic acid and sucrose, and the role of malic acid in pH regulation seemed to suggest an ethylene-induced upregulation of different members of the invertase family activated by the changes in pH, and/or an upregulation of invertase members not localized in the cytoplasm (e.g. cell wall invertase). All these changes in water loss, respiration, and variations of fruit pH and reducing sugars can be linked to the progression of senescence (Alkan and Fortes, 2015). Taken together, the data supports the role of ethylene in promoting senescence in strawberry fruit.

It is well documented that the ABA/auxins ratio plays a major role in regulating strawberry fruit ripening (Iannetta et al., 2006; Jia et al., 2011; Symons et al., 2012; Chen et al., 2015; Chen et al., 2016; Medina-Puche et al., 2016; Moya-León et al., 2019); despite this, there is a lack of information about the role of ABA during storage (Terry et al., 2007a). Results of this study highlighted that continuous exposure to exogenous ethylene did not affect ABA and auxins metabolism in achenes. In contrast, ethylene induced a marked increase in ABA content in the receptacle as a first response (within 24 h), which was sustained through storage (50% higher ABA content than untreated fruit at the end of the storage). In the same tissue, auxins and IAAsp levels were too low to be detectable, and the ABA metabolite 7'OH-ABA did not show any response to ethylene exposure. A study on hormonal content in different strawberry fruit tissues during ripening reported higher ABA amounts in the achenes than in the receptacle (Symons et al., 2012). However, the more recent work of Gu and colleagues (2019) highlighted that ABA content in the receptacle markedly increased with the progression of the ripening and reached a maximum level only at the red stage. The presented data confirmed that in ripe (red) strawberry, ABA concentration 4-times higher in the receptacle tissue compared to achenes through storage. Furthermore, continuous ethylene exposure induced a constant upregulation of the ABA receptacle content (1.24-fold) compared to the control. These findings seemed to suggest a synergistic action of ethylene and ABA: ethylene-induced disequilibrium in the receptacle ABA homeostasis might be a trigger for, and participate in, the regulation of the senescence-related changes observed during storage.

Li and colleagues (2014) described that following UV-C stress, ABA can induce ethylene biosynthesis in unripe detached strawberry, and that ABA levels were inversely correlated to ethylene content. In contrast, the presented data showed a positive correlation between ethylene exposure and ABA content. Moreover, the increasing trend exhibited by ABA throughout storage indicated that the selected ethylene concentration (50 µl l^−1^) was able to keep a positive regulation on ABA accumulation in the receptacle tissue.

ABA is well-known to be also involved in water stress, and following deficit irrigation an increase in phenolic compounds was found in strawberry fruit (Terry et al., 2007b; Weber et al., 2016). Accordingly, samples exposed to ethylene showed higher weight loss, higher ABA content, and higher phenolics accumulation than untreated fruit. This positive effect of ABA on the flavonoid/phenylpropanoid pathway has been explained with the ABA-induced upregulation of *FaSCL8* (*SCARECROW-LIKE 8*) and *FaMYB10* expression (Medina-Puche et al., 2014; Pillet et al., 2015). These two genes have been proposed as key elements for the flavonoid/phenylpropanoid pathway regulation during the ripening of strawberry fruit. The results of the present study showed that epicatechin and chlorogenic acid content were positively affected by ethylene supplementation. Moreover, the sharp increase found in chlorogenic acid at day 1 of storage, and during storage resembled the trend found in ABA content following ethylene exposure. This seemed to support the idea that there is interplay between ethylene and ABA, and that both phytohormones regulate strawberry ripening/senescence during postharvest (**Supplemental Figure 3**).

In conclusion, in this study it is proposed that strawberry postharvest senescent decline is induced by ethylene through ABA action in the receptacle tissue, and that a better understanding of the role of ethylene and ABA and their interplay in mediating senescence would allow better design of shelf life management strategies.

## Data Availability Statement

Data underlying this paper can be accessed at https://doi.org/10.17862/cranfield.rd.11876544.v1.

## Author Contributions

LT conceived and designed the experiments. FE, IP, and KC performed the experiments. RT performed data analysis and wrote the manuscript with the help of LT. All authors contributed to the discussion, revised and approved the final manuscript.

## Funding

Engineering and Physical Sciences Research Council (EPSRC) and Johnson Matthey Plc (case number: 09000987).

## Conflict of Interest

The authors declare that the research was conducted in the absence of any commercial or financial relationships that could be construed as a potential conflict of interest.

The reviewer AL declared a past co-authorship with one of the authors LT to the handling Editor.

## Supplementary Material

Supplemental Figure 1 The effect of ethylene (continuous exposure) on total anthocyanins and pelargonidin-3-glucoside content (mg kg^-1^) of strawberries, cv. Sonata, stored at 5˚ C for 6 d. The represented values show the mean (control and ethylene treated fruit) content. Vertical bar represents LSD (p < 0.05) for the overall means of the significant variable “Storage day”.

Supplemental Figure 2 The effect of ethylene (continuous exposure) on endogenous ethylene production (x10^-11^ g kg^-1^ s^-1^) of strawberries, cv. Sonata, stored at 5˚ C for 6 d. Vertical bar represents LSD (p < 0.05) for the overall means of the significant variable “Storage day”.

Supplemental Figure 3 Graphical abstract of ethylene (continuous exposure) effect on senescence in receptacle and achene tissues in strawberry fruit.
