# Supplementary figures and images for: Continuous Exposure to Ethylene Differentially Affects Senescence in Receptacle and Achene Tissues in Strawberry Fruit
Source: Front Plant Sci. 2020 Mar 12;11:174. doi: 10.3389/fpls.2020.00174 (PMC7080867; doi:10.3389/fpls.2020.00174)

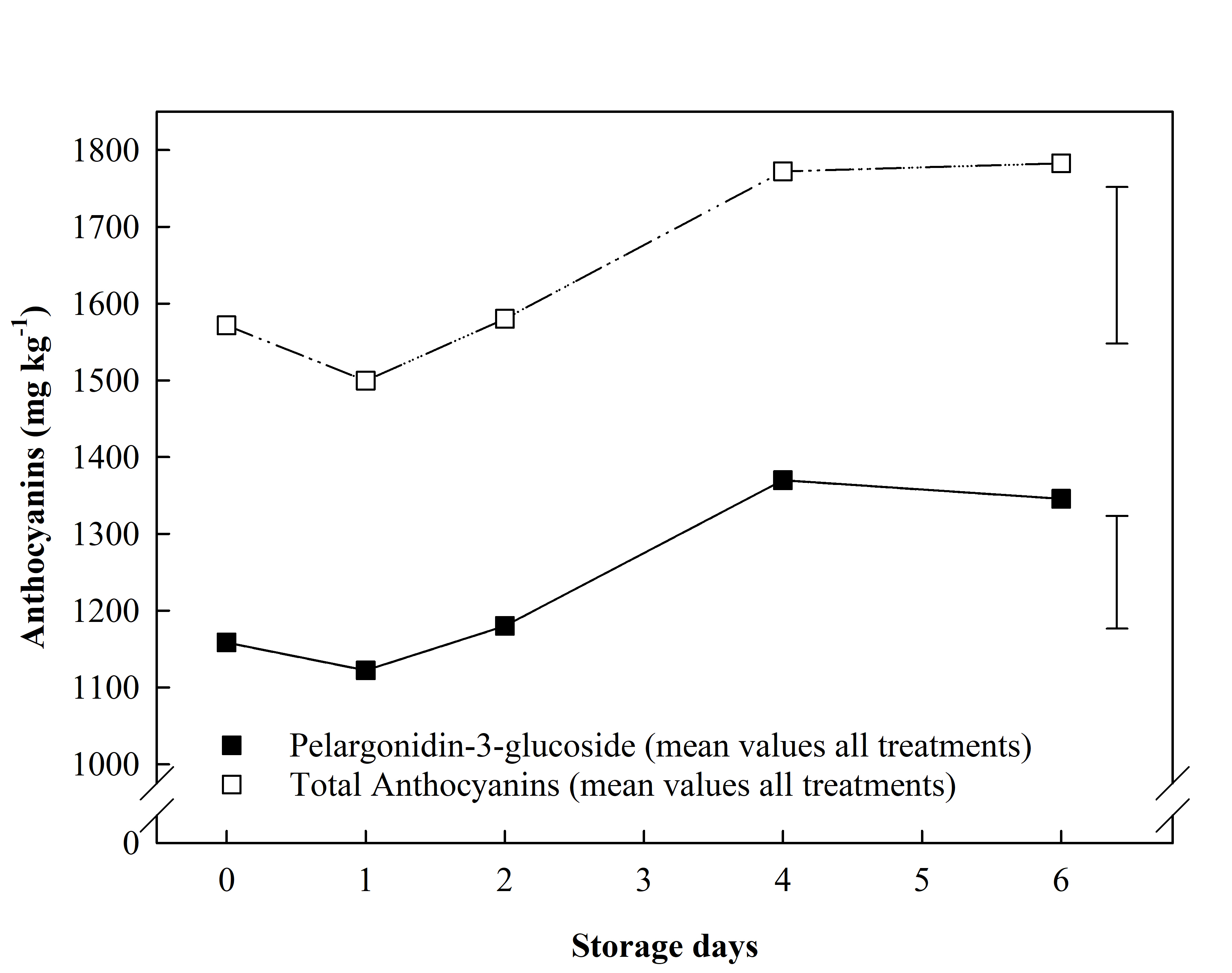

Supplement: Supplemental Figure 1 — The effect of ethylene (continuous exposure) on total anthocyanins and pelargonidin-3-glucoside content (mg kg-1) of strawberries, cv. Sonata, stored at 5˚ C for 6 d. The represented values show the mean (control and ethylene treated fruit) content. Vertical bar represents LSD (p < 0.05) for the overall means of the significant variable “Storage day”. [file Image_1.jpeg]

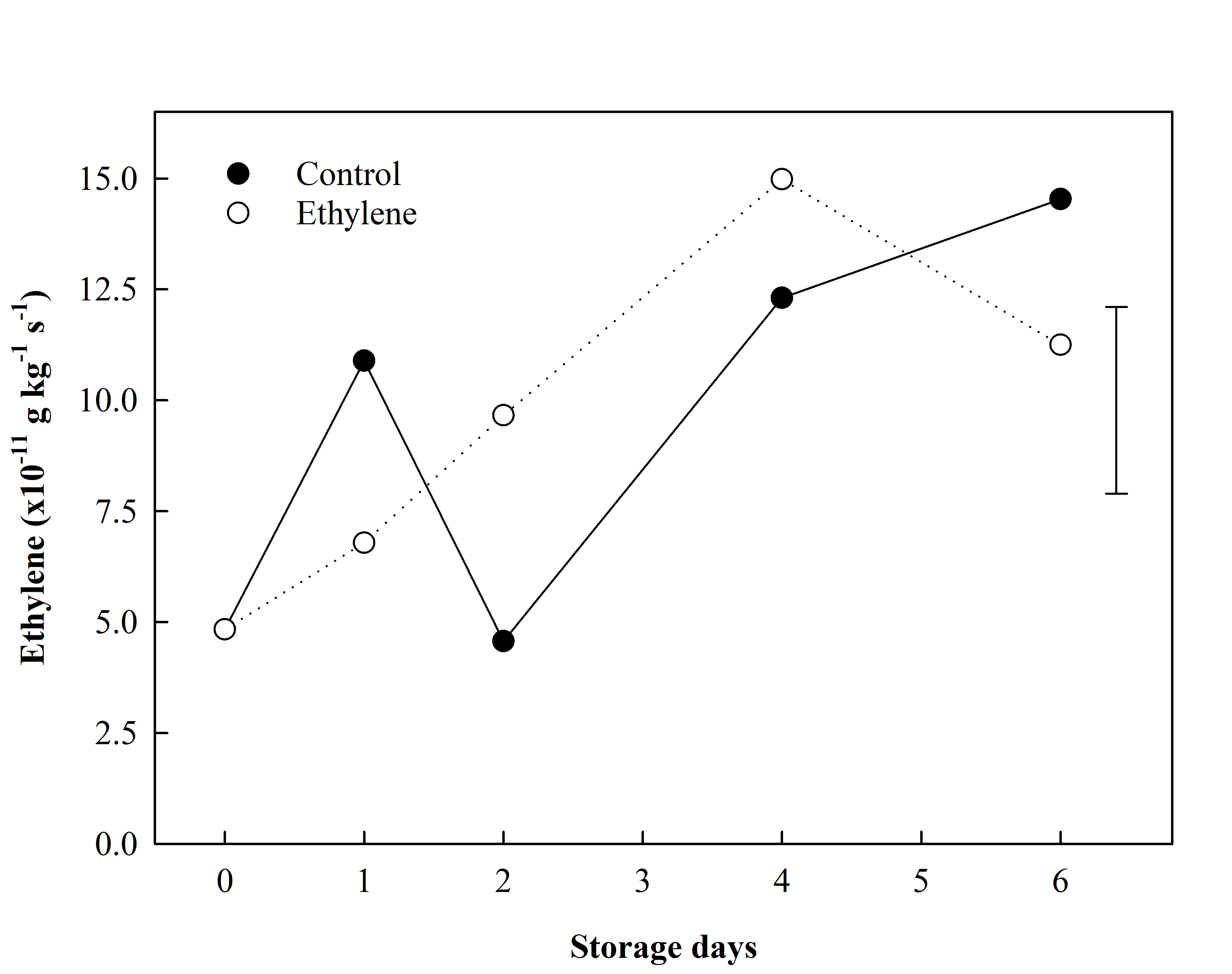

Supplement: Supplemental Figure 2 — The effect of ethylene (continuous exposure) on endogenous ethylene production (x10-11 g kg-1 s-1) of strawberries, cv. Sonata, stored at 5˚ C for 6 d. Vertical bar represents LSD (p < 0.05) for the overall means of the significant variable “Storage day”. [file Image_2.jpeg]

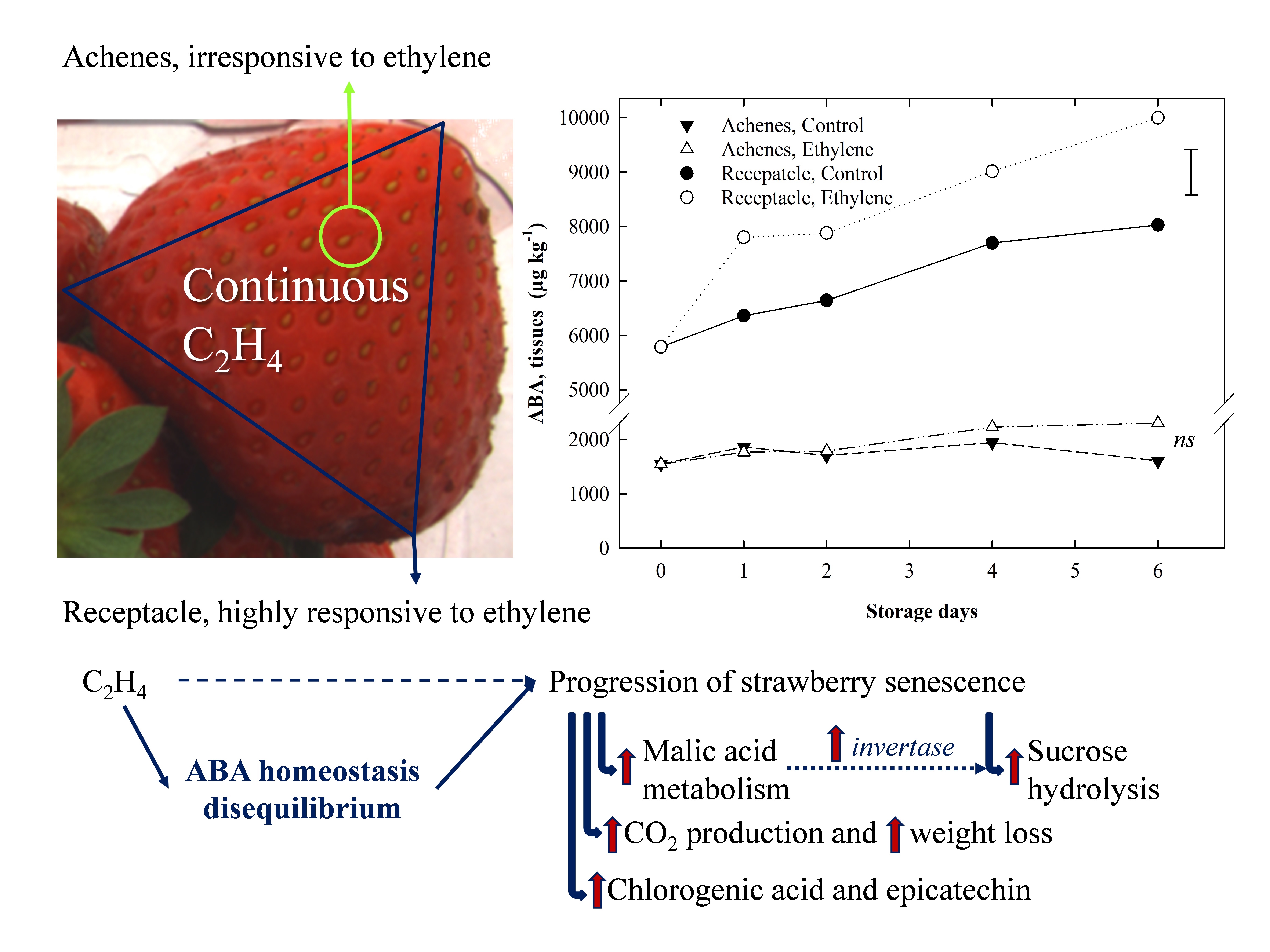

Supplement: Supplemental Figure 3 — Graphical abstract of ethylene (continuous exposure) effect on senescence in receptacle and achene tissues in strawberry fruit. [file Image_3.jpg]
